# *N*,*N*′-Bis(3-chloro-2-fluoro­benzyl­idene)ethane-1,2-diamine

**DOI:** 10.1107/S1600536808028419

**Published:** 2008-09-13

**Authors:** Hoong-Kun Fun, Reza Kia

**Affiliations:** a X-ray Crystallography Unit, School of Physics, Universiti Sains Malaysia, 11800 USM, Penang, Malaysia

## Abstract

The mol­ecule of the title centrosymmetric Schiff base compound, C_16_H_12_Cl_2_F_2_N_2_, adopts an *E* configuration with respect to the azomethine C=N bond. The imino groups are coplanar with the aromatic rings. Within the mol­ecule, the planar units are parallel, but extend in opposite directions from the dimethyl­ene bridge. An inter­esting feature of the crystal structure is the short inter­molecular Cl⋯F [3.1747 (5) Å] inter­actions, which are shorter than the sum of the van der Waals radii of these atoms. These inter­actions link neighbouring mol­ecules along the *b* axis. The crystal structure is further stabilized by π–π inter­actions, with a centroid–centroid distance of 3.5244 (4) Å.

## Related literature

For bond-length data, see Allen *et al.* (1987 ▶). For related structures, see, for example: Fun & Kia (2008*a* ▶,*b* ▶): Fun, Kargar & Kia (2008 ▶); Fun, Kia & Kargar (2008 ▶). For information on Schiff base complexes and their applications, see, for example: Pal *et al.* (2005 ▶); Calligaris & Randaccio (1987 ▶); Hou *et al.* (2001 ▶); Ren *et al.* (2002 ▶). For hydrogen-bonding motifs, see: Bernstein *et al.* (1995 ▶).
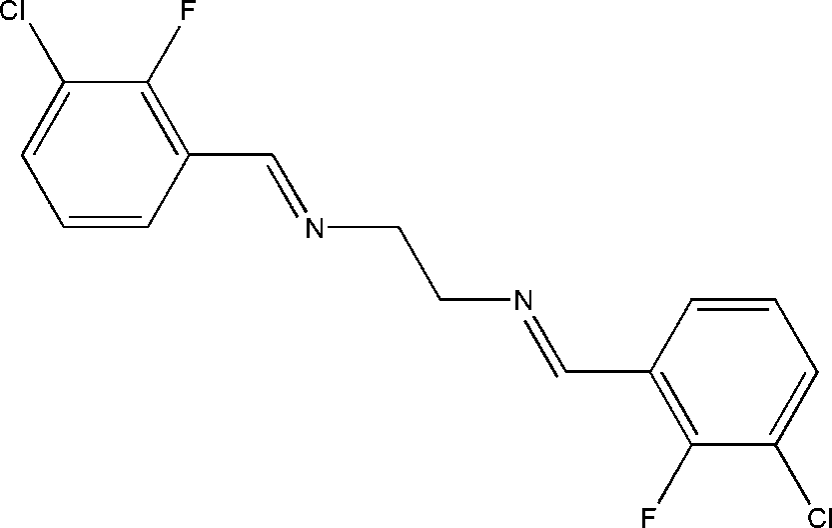


## Experimental

### 

#### Crystal data

C_16_H_12_Cl_2_F_2_N_2_
*M* *_r_* = 341.18
Monoclinic, 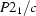

*a* = 7.2249 (2) Å
*b* = 11.3676 (2) Å
*c* = 10.3368 (2) Å
β = 120.906 (1)°
*V* = 728.42 (3) Å^3^
*Z* = 2
Mo *K*α radiation
μ = 0.46 mm^−1^
*T* = 100.0 (1) K
0.52 × 0.41 × 0.29 mm

#### Data collection

Bruker SMART APEXII CCD area-detector diffractometer
Absorption correction: multi-scan (*SADABS*; Bruker, 2005 ▶) *T*_min_ = 0.794, *T*_max_ = 0.878
16842 measured reflections
3821 independent reflections
3403 reflections with *I* > 2σ(*I*)
*R*_int_ = 0.025

#### Refinement

*R*[*F* ^2^ > 2σ(*F* ^2^)] = 0.030
*wR*(*F* ^2^) = 0.088
*S* = 1.05
3821 reflections
100 parameters
H-atom parameters constrained
Δρ_max_ = 0.45 e Å^−3^
Δρ_min_ = −0.35 e Å^−3^

### 

Data collection: *APEX2* (Bruker, 2005 ▶); cell refinement: *APEX2*; data reduction: *SAINT* (Bruker, 2005 ▶); program(s) used to solve structure: *SHELXTL* (Sheldrick, 2008 ▶); program(s) used to refine structure: *SHELXTL*; molecular graphics: *SHELXTL*; software used to prepare material for publication: *SHELXTL* and *PLATON* (Spek, 2003 ▶).

## Supplementary Material

Crystal structure: contains datablocks global, I. DOI: 10.1107/S1600536808028419/sj2537sup1.cif

Structure factors: contains datablocks I. DOI: 10.1107/S1600536808028419/sj2537Isup2.hkl

Additional supplementary materials:  crystallographic information; 3D view; checkCIF report

## Figures and Tables

**Fig. 1.**
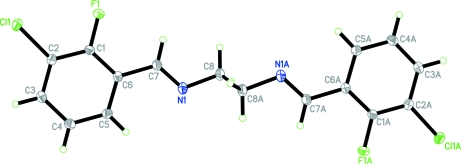
The molecular structure of (I) with atom labels and 50% probability ellipsoids for non-H atoms. The suffix A corresponds to symmetry code (-x + 2, -y, -z + 1).

**Fig. 2.**
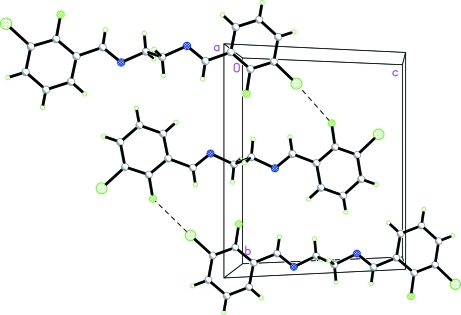
The crystal packing of (I), viewed approximately down the a-axis, showing the linking of the molecules by Cl···F contacts along the b-axis. Intermolecular interactions are shown as dashed lines.

## supplementary crystallographic information

### Comment

Schiff bases are among the most prevalent mixed-donor ligands in the field of coordination chemistry in which there has been growing interest, mainly because of their wide application in areas such as biochemistry, synthesis, and catalysis (Pal *et al.*, 2005; Hou *et al.*, 2001; Ren *et al.*, 2002). Many Schiff base complexes have been structurally characterized, but only a relatively small number of free Schiff bases have had their X-ray structures reported (Calligaris & Randaccio, 1987). As an extension of our work (Fun, Kargar & Kia 2008; Fun, Kia & Kargar 2008) on the structural characterization of Schiff base ligands, the title compound (I), is reported here.

The molecule of the title compound (Fig. 1), adopts an *E* configuration with respect to the azomethine C═N bond. The bond lengths (Allen *et al.*, 1987) and angles are within normal ranges and are comparable with the values found in related structures (Fun & Kia 2008*a*,*b*; Fun, Kargar & Kia 2008; Fun, Kia & Kargar 2008). The two planar units are parallel but extend in opposite directions from the dimethylene bridge. The interesting feature of the crystal structure is the short intermolecular Cl···F interactions [symmetry code: *x*, -1/2 - *y*, -1/2 + *z*] with a distance of 3.1747 (5) Å, which is shorter than the sum of the van der Waals radii of these atoms. These interactions link neighbouring molecules along the *b*-axis. The crystal structure is further stabilized by π–π interactions with a centroid to centroid distance of 3.5244 (4) Å [*Cg*1–*Cg*1; symmetry code, 2 - *x*, -*y*, -*z*; *Cg*1 is the centroid of the C1–C6 benzene ring].

### Experimental

The synthetic method has been described earlier (Fun, Kargar & Kia, 2008). Single crystals suitable for X-ray diffraction were obtained by evaporation of an ethanol solution at room temperature.

### Refinement

All of the hydrogen atoms were positioned geometrically with C—H = 0.95 or 0.99 Å and refined in riding mode with *U*_iso_ (H) = 1.2 *U*_eq_ (C).

### Crystal data

**Table:**

C_16_H_12_Cl_2_F_2_N_2_	*F*(000) = 348
*M**_r_* = 341.18	*D*_x_ = 1.556 Mg m^−^^3^
Monoclinic, *P*2_1_/*c*	Mo *K*α radiation, λ = 0.71073 Å
Hall symbol: -P 2ybc	Cell parameters from 8904 reflections
*a* = 7.2249 (2) Å	θ = 2.9–41.0°
*b* = 11.3676 (2) Å	µ = 0.46 mm^−^^1^
*c* = 10.3368 (2) Å	*T* = 100 K
β = 120.906 (1)°	Block, colourless
*V* = 728.42 (3) Å^3^	0.52 × 0.41 × 0.29 mm
*Z* = 2	

### Data collection

**Table:**

Bruker SMART APEXII CCD area-detector diffractometer	3821 independent reflections
Radiation source: fine-focus sealed tube	3403 reflections with *I* > 2σ(*I*)
graphite	*R*_int_ = 0.025
φ and ω scans	θ_max_ = 37.5°, θ_min_ = 2.9°
Absorption correction: multi-scan (SADABS; Bruker, 2005)	*h* = −11→12
*T*_min_ = 0.794, *T*_max_ = 0.878	*k* = −19→19
16842 measured reflections	*l* = −17→17

### Refinement

**Table:**

Refinement on *F*^2^	Primary atom site location: structure-invariant direct methods
Least-squares matrix: full	Secondary atom site location: difference Fourier map
*R*[*F*^2^ > 2σ(*F*^2^)] = 0.030	Hydrogen site location: inferred from neighbouring sites
*wR*(*F*^2^) = 0.088	H-atom parameters constrained
*S* = 1.05	*w* = 1/[σ^2^(*F*_o_^2^) + (0.0499*P*)^2^ + 0.131*P*] where *P* = (*F*_o_^2^ + 2*F*_c_^2^)/3
3821 reflections	(Δ/σ)_max_ = 0.001
100 parameters	Δρ_max_ = 0.45 e Å^−^^3^
0 restraints	Δρ_min_ = −0.35 e Å^−^^3^

### Special details

**Table:**

Experimental. The low-temperature data was collected with the Oxford Cyrosystem Cobra low-temperature attachment.
Geometry. All e.s.d.'s (except the e.s.d. in the dihedral angle between two l.s. planes) are estimated using the full covariance matrix. The cell e.s.d.'s are taken into account individually in the estimation of e.s.d.'s in distances, angles and torsion angles; correlations between e.s.d.'s in cell parameters are only used when they are defined by crystal symmetry. An approximate (isotropic) treatment of cell e.s.d.'s is used for estimating e.s.d.'s involving l.s. planes.
Refinement. Refinement of *F*^2^ against ALL reflections. The weighted *R*-factor *wR* and goodness of fit *S* are based on *F*^2^, conventional *R*-factors *R* are based on *F*, with *F* set to zero for negative *F*^2^. The threshold expression of *F*^2^ > σ(*F*^2^) is used only for calculating *R*-factors(gt) *etc*. and is not relevant to the choice of reflections for refinement. *R*-factors based on *F*^2^ are statistically about twice as large as those based on *F*, and *R*- factors based on ALL data will be even larger.

### Fractional atomic coordinates and isotropic or equivalent isotropic displacement parameters (Å^2^)

**Table:**

	*x*	*y*	*z*	*U*_iso_*/*U*_eq_	
Cl1	0.65534 (3)	−0.131316 (15)	−0.342553 (18)	0.01852 (5)	
F1	0.71054 (8)	−0.18319 (4)	−0.04997 (5)	0.01952 (9)	
N1	0.83760 (10)	0.03597 (6)	0.29627 (6)	0.01620 (10)	
C1	0.72180 (10)	−0.06703 (6)	−0.07109 (7)	0.01350 (10)	
C2	0.69340 (10)	−0.02919 (6)	−0.20779 (7)	0.01388 (10)	
C3	0.70125 (11)	0.09019 (6)	−0.23283 (7)	0.01577 (11)	
H3	0.6802	0.1170	−0.3265	0.019*	
C4	0.74016 (11)	0.17052 (6)	−0.11975 (8)	0.01704 (11)	
H4	0.7457	0.2523	−0.1363	0.020*	
C5	0.77080 (11)	0.13130 (6)	0.01692 (8)	0.01554 (11)	
H5	0.7977	0.1868	0.0934	0.019*	
C6	0.76267 (10)	0.01123 (6)	0.04403 (7)	0.01320 (10)	
C7	0.80350 (10)	−0.03353 (6)	0.19019 (7)	0.01486 (11)	
H7	0.8043	−0.1160	0.2053	0.018*	
C8	0.88630 (11)	−0.01757 (7)	0.43842 (7)	0.01710 (11)	
H8A	0.8762	−0.1043	0.4279	0.021*	
H8B	0.7805	0.0092	0.4659	0.021*	

### Atomic displacement parameters (Å^2^)

**Table:**

	*U*^11^	*U*^22^	*U*^33^	*U*^12^	*U*^13^	*U*^23^
Cl1	0.02300 (8)	0.01901 (9)	0.01500 (8)	−0.00281 (5)	0.01081 (6)	−0.00347 (5)
F1	0.0292 (2)	0.01227 (18)	0.01890 (19)	−0.00308 (15)	0.01361 (17)	0.00007 (14)
N1	0.0180 (2)	0.0181 (2)	0.0121 (2)	0.00091 (18)	0.00742 (18)	0.00082 (17)
C1	0.0142 (2)	0.0125 (2)	0.0135 (2)	−0.00087 (18)	0.00691 (18)	0.00018 (18)
C2	0.0139 (2)	0.0153 (3)	0.0124 (2)	−0.00011 (18)	0.00672 (18)	−0.00027 (18)
C3	0.0166 (2)	0.0165 (3)	0.0141 (2)	0.0015 (2)	0.0079 (2)	0.00275 (19)
C4	0.0201 (3)	0.0140 (3)	0.0170 (2)	0.0024 (2)	0.0096 (2)	0.0025 (2)
C5	0.0183 (3)	0.0131 (3)	0.0151 (2)	0.00170 (19)	0.0085 (2)	0.00006 (18)
C6	0.0133 (2)	0.0140 (2)	0.0121 (2)	0.00054 (18)	0.00633 (18)	0.00056 (18)
C7	0.0158 (2)	0.0161 (3)	0.0122 (2)	−0.00085 (19)	0.00683 (19)	0.00053 (19)
C8	0.0178 (2)	0.0212 (3)	0.0122 (2)	−0.0012 (2)	0.0076 (2)	0.0008 (2)

### Geometric parameters (Å, °)

**Table:**

Cl1—C2	1.7235 (7)	C4—C5	1.3865 (10)
F1—C1	1.3476 (8)	C4—H4	0.9500
N1—C7	1.2687 (9)	C5—C6	1.4007 (9)
N1—C8	1.4568 (9)	C5—H5	0.9500
C1—C2	1.3878 (9)	C6—C7	1.4733 (9)
C1—C6	1.3906 (9)	C7—H7	0.9500
C2—C3	1.3882 (9)	C8—C8^i^	1.5267 (13)
C3—C4	1.3934 (10)	C8—H8A	0.9900
C3—H3	0.9500	C8—H8B	0.9900
			
C7—N1—C8	116.79 (6)	C4—C5—H5	119.5
F1—C1—C2	118.62 (6)	C6—C5—H5	119.5
F1—C1—C6	119.48 (6)	C1—C6—C5	117.68 (6)
C2—C1—C6	121.90 (6)	C1—C6—C7	119.95 (6)
C1—C2—C3	119.61 (6)	C5—C6—C7	122.33 (6)
C1—C2—Cl1	119.54 (5)	N1—C7—C6	121.26 (6)
C3—C2—Cl1	120.84 (5)	N1—C7—H7	119.4
C2—C3—C4	119.62 (6)	C6—C7—H7	119.4
C2—C3—H3	120.2	N1—C8—C8^i^	109.32 (7)
C4—C3—H3	120.2	N1—C8—H8A	109.8
C5—C4—C3	120.12 (6)	C8^i^—C8—H8A	109.8
C5—C4—H4	119.9	N1—C8—H8B	109.8
C3—C4—H4	119.9	C8^i^—C8—H8B	109.8
C4—C5—C6	121.07 (6)	H8A—C8—H8B	108.3
			
F1—C1—C2—C3	179.08 (6)	C2—C1—C6—C5	1.11 (9)
C6—C1—C2—C3	−1.35 (10)	F1—C1—C6—C7	2.90 (9)
F1—C1—C2—Cl1	−2.49 (8)	C2—C1—C6—C7	−176.66 (6)
C6—C1—C2—Cl1	177.08 (5)	C4—C5—C6—C1	−0.31 (10)
C1—C2—C3—C4	0.78 (10)	C4—C5—C6—C7	177.39 (6)
Cl1—C2—C3—C4	−177.63 (5)	C8—N1—C7—C6	−177.18 (6)
C2—C3—C4—C5	−0.01 (10)	C1—C6—C7—N1	−178.79 (6)
C3—C4—C5—C6	−0.22 (11)	C5—C6—C7—N1	3.55 (10)
F1—C1—C6—C5	−179.33 (6)	C7—N1—C8—C8^i^	117.01 (8)

Symmetry codes: (i) −*x*+2, −*y*, −*z*+1.
